# Comprehensive Network Analysis Identified SIRT7, NTRK2, and CHI3L1 as New Potential Markers for Intervertebral Disc Degeneration

**DOI:** 10.1155/2022/4407541

**Published:** 2022-02-12

**Authors:** Haoxi Li, Wenhao Li, Li Zhang, Jicheng He, Lin Tang, Zhuhai Li, Feng Chen, Qie Fan, Jianxun Wei

**Affiliations:** ^1^Department of Spine Surgery, The People's Hospital of Guangxi Zhuang Autonomous Region, Nanning 530021, China; ^2^Department of Pathology, The Third People's Hospital of Guangxi Zhuang Autonomous Region, Nanning 530021, China

## Abstract

Intervertebral disc degeneration (IDD) is considered the basis of serious clinical symptoms, especially for low back pain (LBP). Therefore, it is essential to explore the regulatory role and diagnostic performance of dysregulated genes and potential drugs in IDD. Through WGCNA co-expression analysis, 36 co-expression modules were obtained. Among them, MidnightBlue and Red modules were the most related to IDD. Functional enrichment analysis showed that the Red module was mainly related to neutrophil activation and regulation of cytokine-mediated signaling pathway and apoptosis, whereas the MidnightBlue module was mainly related to extracellular matrix organization, bone development, extracellular matrix, extracellular matrix component, and other extracellular matrices. Furthermore, 356 genes highly related to the module were screened to construct a protein interaction network. Network degree distribution analysis showed that the known IDD-related genes had a higher degree of distribution. Enrichment analysis demonstrated that these genes were enriched in MAPK_SIGNALING_PATHWAY (FDR = 0.012), CHEMOKINE_SIGNALING_PATHWAY, and some other pathways. By constructing a disease-gene interaction network, three disease-specific genes were finally identified. Through combining with the drug-target gene interaction network, two potential therapeutic drugs, entrectinib and larotrectinib, were determined. Finally, based on these genes, the diagnostic model in the training dataset, test dataset, and verification dataset all showed a high diagnostic performance. The findings of this study contributed to the diagnosis of IDD and personalized treatment of IDD.

## 1. Introduction

Low back pain (LBP) is a multifactor disease, with intervertebral disc degeneration (IDD) as a main causal factor [1]. The aging of process intervertebral disc [2] will lead to the degeneration of vertebral disc (IVD), resulting in nerve symptoms including LBP [3]; 80% of the world population was reported to suffer from LBP, which could even cause the loss of labor in severe cases [4, 5]. Due to the lack of a clear understanding of the pathological mechanism of IDD, treatment or delay of IDD seems to be ineffective. With the aging of the population, the incidence of IDD-induced LBP is further increasing, pointing to the need of exploring the pathological mechanism of IDD.

Large-scale gene expression studies showed that many coding genes are differentially expressed in IDD, and some of them play an important role in IDD [6, 7]. For example, the expression of the inflammation-associated autocrine factor CHI3L1, a tissue specific in nucleus pulposus (NP), is significantly upregulated during denaturation, and this protects IDD by promoting the Akt3 signaling pathway [8]. With the development of genetic and proteomic tools, our understanding of genetic disorders in IDD has greatly improved. Targeted dissonant gene therapy strategies developed encouraging results from animal models of IDD [9]. The novel lentiviral vector expressing CHOP shRNA effectively inhibits the apoptosis of rat annulus fibrosus (AF) cells by silencing the expression of CHOP [10].

In recent years, more and more bioinformatics research has been carried out on disc repair, and some effective analysis results have been obtained. For example, bioinformatic analyses identified CCND1, GATA3, TNFSF11, LEF1, and DKK1 were related to degenerative disc diseases [11]. Based on bioinformatics analysis, LOC102555094 might be demethylated by ZFP217, activating FTO, and LOC102555094/miR-431/GSK-3*β*/Wnt played a crucial role in IDD [12]. Jinwen Zhu et al. identified several lncRNA/circRNA-miRNA-mRNA interaction axes (MALAT1/hsa_circRNA_102348-hsa-miR-185-5p- TGFB1/FOS, MALAT1-hsa-miR-155-5p-HIF1A, hsa_circRNA_102399-hsa-miR-302a-3p-HIF1A, MALAT1-hsa-miR-519d-3p-MAPK1, and hsa_circRNA_100086-hsa-miR-509-3p-MAPK1), which may be crucial for the treatment of IDD [13].

The purpose of this study was to investigate the potential function of mRNA expression in IDD based on RNA expression profiles from IDD patients. We systematically analyzed mRNA expression profiles between IDD and healthy patients. In addition, we developed a novel algorithm for identifying mRNAs during IDD progression to determine mRNA biomarkers for IDD diagnosis and prognosis.

## 2. Results

### 2.1. Identification of IDD-Related Gene Modules

**Figure 1:**
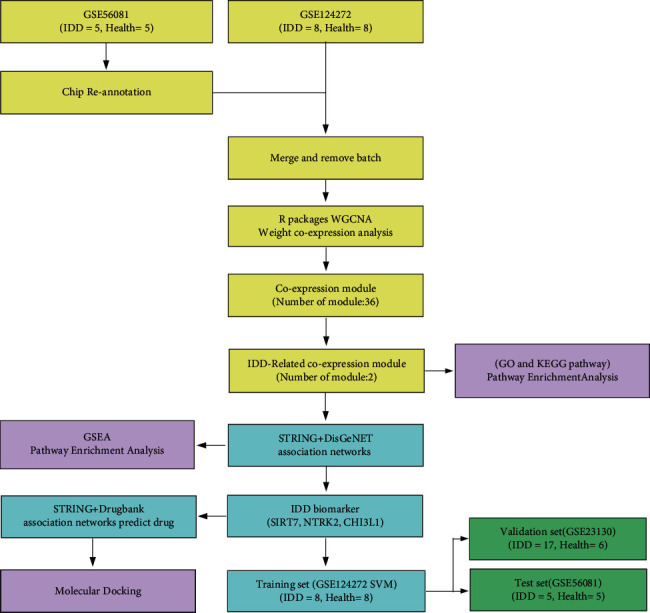
Workflow chart.

**Figure 2:**
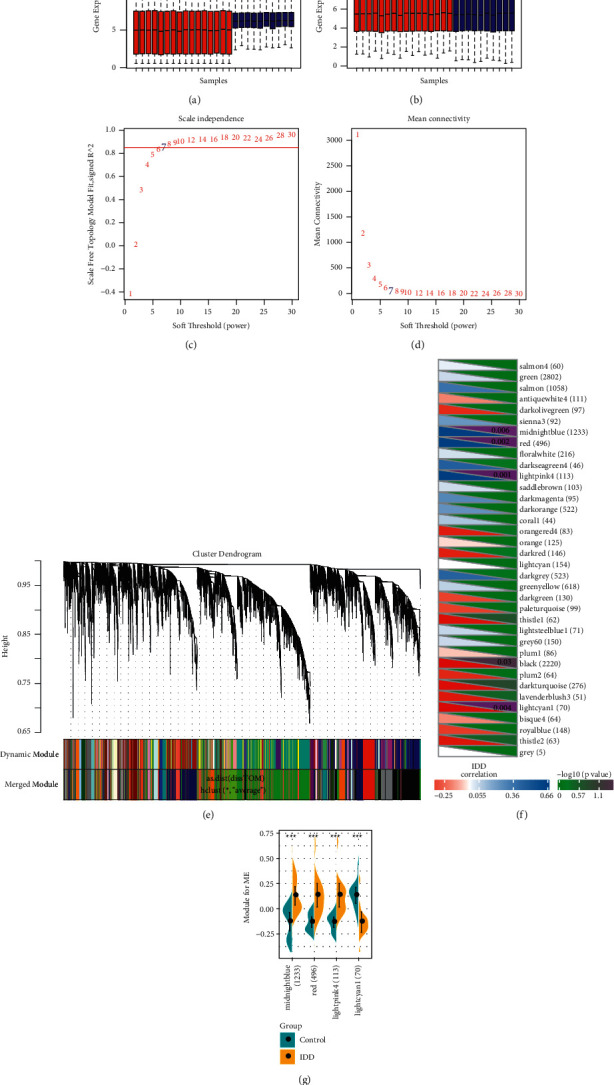
Identification of IDD-related modules. (a) Expression distribution in each sample in the combined dataset of GSE56081 and GSE124272. Blue is the GSE56081 dataset sample, Red is the GSE124272 dataset sample. (b) Expression distribution in each sample in GSE56081 and GSE124272 datasets after removing batch effect, Blue is the GSE56081 dataset sample, Red is the GSE124272 dataset sample. (c) Analysis of the scale-free fit index for various soft-thresholding powers (*β*). (d) Analysis of the mean connectivity for various soft-thresholding powers. (e) Dendrogram of all expressed genes clustered based on a dissimilarity measure (1-TOM). (f) The correlation between co-expression module and IDD, where the upper right corner represents significant *P* value, and the lower left corner represents correlation coefficient, ^*∗*^*p* < 0.05, ^*∗∗*^*p* < 0.01, and ^*∗∗∗*^*p* < 0.001. The number in parentheses is the number of genes in the module. (g) The difference distribution of the feature vectors of modules that are significantly related to IDD in IDD and the control group.

Methodology consisted of data collection, batch effect removal, co-expression module identification, and enrichment analysis, followed by protein network construction, network feature selection, and classifier construction and verification. The workflow is shown in Figure 1. The datasets GSE56081 and GSE124272 were obtained from GEO, and the data were standardized and re-annotated on the chip. To include more sample sizes, the GSE56081 and GSE124272 expression profile datasets were merged, and finally, we obtained the expression profiles of 12296 genes. The overall gene expression in the GSE56081 dataset was higher than that in the GSE124272 dataset, and there is a batch effect (Figure 2(a)), which was removed using the R software package SVA to obtain a new expression profile. As the new profile showed consistent distribution among the datasets (Figure 2(b)), this suggested that the expression profile without batch effects was qualified for further data analysis. The abnormal gene expression modules in IDD were analyzed by applying the R software package WGCNA to analyze IDD-related co-expression modules based on gene expression profiles. In this study, the power of *β* = 7 (*R*^2 > 0.85 without scale) was the soft threshold to ensure the scale-free network (Figure 2(c) and 2(d)). A total of 36 modules were identified (Figure 2(e)). The correlation between diseases and modules was determined. Firstly, the Pearson correlation coefficient between the feature vectors of each module and the occurrence of diseases was calculated (Figure 2(f)). Further analysis on the distribution difference of the feature vectors of the significantly related modules in IDD and the control group showed that the feature vector distribution of the disease group in LightPink4, MidnightBlue, and Red modules was remarkably higher than that of the healthy group, whereas the feature vector distribution of the LightCyan1 module in the disease group was significantly lower than that of the healthy group (Figure 2(g)). Based on these two methods, LightPink4, MidnightBlue, Red, and LightCyan1 modules, which were found to be closely related to the occurrence of IDD, were determined as the key modules of IDD in this study.

### 2.2. Functional Involvement of IDD-Related Modules

**Figure 3:**
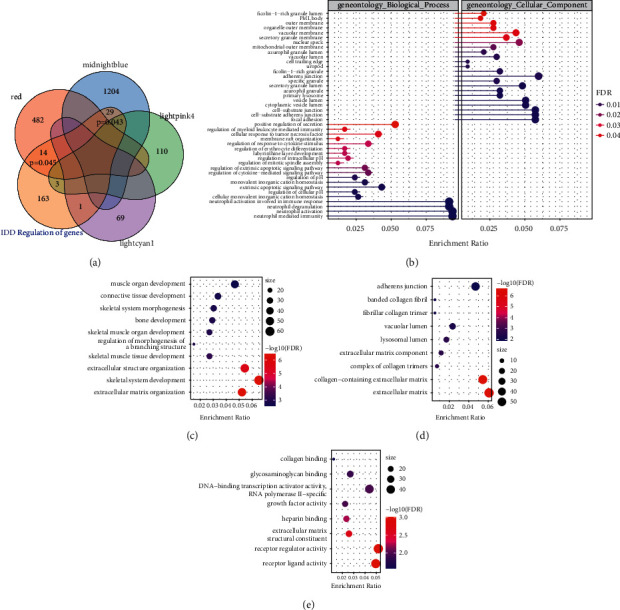
Functional analysis of IDD-related modules. (a) Winn diagram of intersection between genes in four IDD-related modules and known regulatory genes of IDD. (b) GO Biological Process and Cellular Component enriched by genes in the Red module. (c) The most significant 10 bcCellular components enriched by the MidnightBlue module. (c) The most significant 10 molecular functions enriched in the MidnightBlue module. The dot size in the figure represents the number of module genes enriched in term. Color denotes significance FDR.

To better understand the functional involvement of the four disease-related modules, IDD-related genes were first obtained from the DisGeNET [14]. The intersection of gene sets and IDD-related regulatory genes in the four IDD-related modules was analyzed (Figure 3(a)). We found that the genes in Red and MidnightBlue modules showed significant intersection with IDD-related regulatory genes (*P* < 0.05), suggesting that the genes in Red and MidnightBlue modules were biologically correlated with IDD. GO functional enrichment analysis was performed on the Red and MidnightBlue modules. The Red module was enriched to 20 GO biological processes, which are mainly related to neutrophil activation and regulation of cytokine-mediated signaling pathway and apoptosis, and to another 23 cellular components that mainly involved cellular outer membrane and cell adhesion (Figure 3(b)). Similarly, the MidnightBlue module was enriched to a large number of GO terms but most significantly to 10 biological processes, which mainly included extracellular matrix organization, bone development, and other biological processes (Figure 3(c)). The top 10 cellular components contained extracellular matrix, extracellular matrix component, and other components related to extracellular matrix (Figure 3(d)). In addition, the MidnightBlue module was also enriched in many molecular functions, such as receptor regulator activity and extracellular matrix structural constituent (Figure 3(e)). Previous reports indicated that pro-inflammatory cytokines, immune cells secretion, and cytokines regulate extracellular matrix in the intervertebral disc-abnormal modification enzymes, causing an imbalance between metabolic enzymes and anabolic enzymes, which will lead to widespread back, neck and back pain [15]. These results suggested genes in the Red and MidnightBlue modules shared a strong biological correlation with IDD.

### 2.3. Construction of IDD-Specific Protein Interaction Network

**Figure 4:**
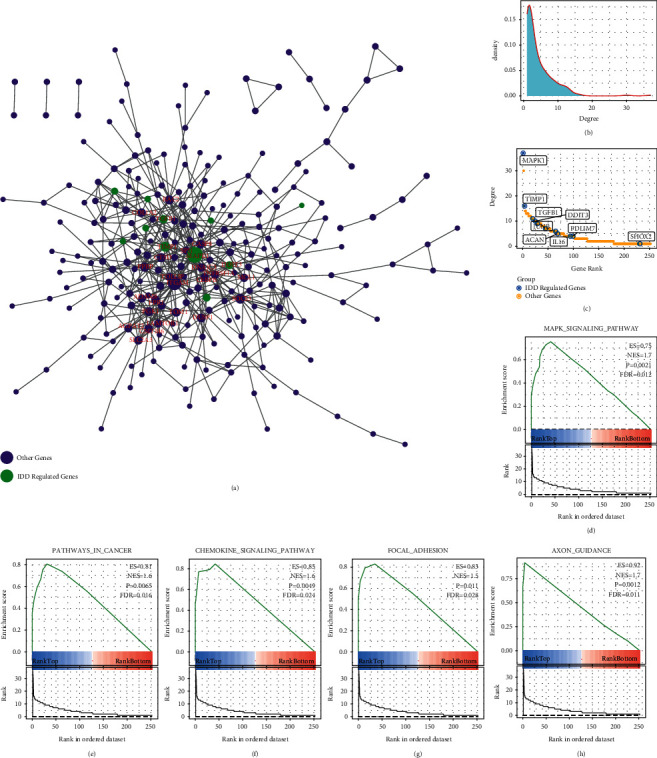
Analysis of IDD-specific protein interaction networks. (a) IDD-specific protein interaction networks. (b) Degree distribution of the network. (c) Degree rank of each node in the network, marked as IDDRG; D-H: the KEGG pathway is enriched by ranked GSEAs in the network.

To identify new IDD-related genes, the gene sets in the Red and MidnightBlue modules were selected, and the Pearson correlations between the genes in the modules and the feature vectors of the modules were calculated, respectively. A total of 855 genes with a correlation greater than 0.7 were selected, and the expression table of these genes was further calculated to determine the AUC of IDD. We obtained a total of 356 genes with AUC higher than 0.8 and mapped these 356 genes to the STRING database [16] (https://string-db.org/). From here, 533 interaction data involving 252 genes were collected to construct an IDD-specific protein interaction network. In the network, a few genes were linked by a large number of other genes, and many genes only interacted with a few genes (Figure 4(a)), and among these genes, MAPK1 was relatively the genes with the largest interaction with other genes in the network. The p38 MAPK signaling pathway plays an important role in many inflammatory and metabolic changes during disc degeneration [17]. The degree distribution in the network was analyzed (Figure 4(b)), and it has been found that the majority of nodes had degrees around 1 and a few nodes were above 10, showing a median law distribution, which is consistent with the characteristics of biological networks. There were 9 known IDD-regulated genes in the network, and most of these genes had a large degree ranking, suggesting that a larger node degree in the IDD-specific protein interaction network is more closely related to IDD (Figure 4(c)). The degree of nodes in the network was used as rank for GSEA function enrichment analysis, and these genes were found to be significantly enriched into 5 KEGG pathways (Figure 4(d)–4(h)), which were MAPK_SIGNALING_Pathway (FDR = 0.012) and CHEMOKINE_SIGNALING_Pathway (FDR = 0.024).

### 2.4. Key Genes of IDD Were Mined and Identified

**Figure 5:**
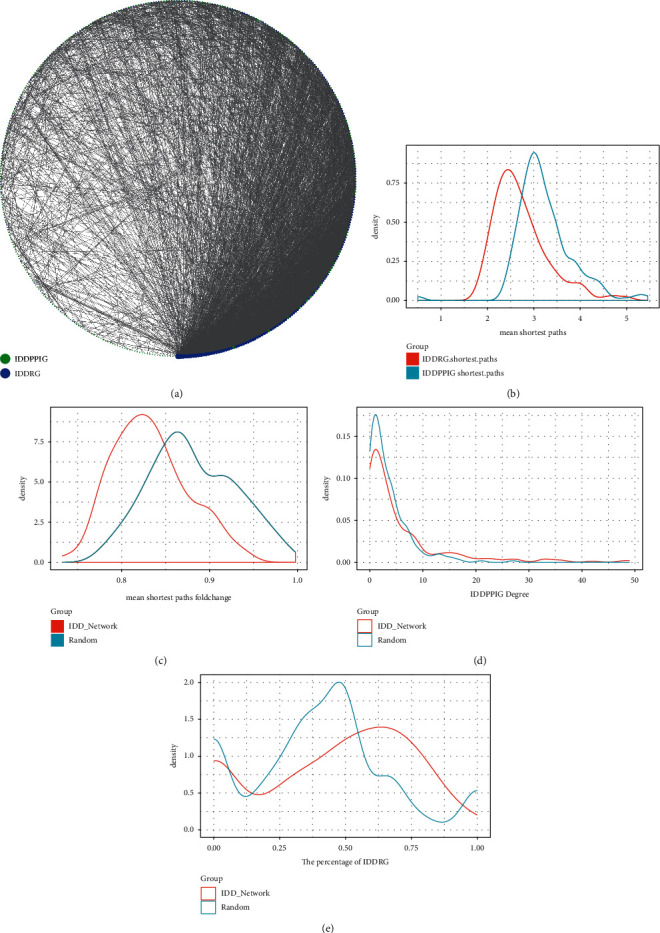
Integrated analysis of IDD-specific protein network and IDD-regulated gene network. (a) Interaction network of IDDRG and IDDPPIG. (b) The average shortest path distribution between IDDPPIG and IDDRG and other IDDPPIG in the network. (c) Distribution of the average shortest path multiples between IDDPPIG and IDDRG and other IDDPPIG in the network. (d) Degree distribution of IDDPPIG in the network. (e) IDDRG Proportion Distribution of IDDRG Interaction in the Network.

Considering the significance of IDD-specific protein networks, we introduced all IDDRGs into the network. The interaction relationships between two IDDRGs and between two IDDPPIG were obtained from the STRING database to construct a new IDD regulation network, which contained 435 nodes and 4362 pieces of interaction information, and there were 194 IDDRGs (Figure 5(a)). We found that the degree of IDDRG in the network was significantly higher than that of IDDPIG. The enrichment significance of each IDDPPIG gene by IDDRG was calculated, and the results demonstrated that a total of 168 IDDPPIG genes (69.7%) were significantly enriched by IDDRG with a *P* < 0.05, suggesting that a large number of IDDPPIG genes in the network were indirectly or interrelated with IDDRG. The network characteristics of IDDRG and IDDPPIG were further systematically compared, and it was observed that the average shortest path between each IDDPPIG and IDDRG was significantly (*p*=1*E* − 16) shorter than the average shortest path between other IDDPPIGs (Figure 5(b)), indicating that there was a closer interaction relationship between IDDPPIG and IDDRG. The multiples of the average shortest path from an IDDPPIG gene to an IDDRG and the average shortest path from each IDDPPIG gene to other IDDPPIG were calculated, and we found that most of them were between 0.8 and 0.85, which was lower than that of the random network (Figure 5(c)). After analyzing the degree distribution of each IDDPPIG in the network, it is observed that the average degree was higher than that of the random network (Figure 5(d)). In addition, we also found a higher proportion of IDDRG interacting with IDDPPIG gene than that in the random network (Figure 5(e)).

Based on the above results, IDDRG with a significantly high interaction ratio and IDDPPIG with both significantly low multiple of shortest path and high degree of distribution were selected as a new potential key gene of IDD. Here, we obtained three genes (Table 1).

### 2.5. Potential Drugs and Drug Targets of Key IDD Genes

**Figure 6:**
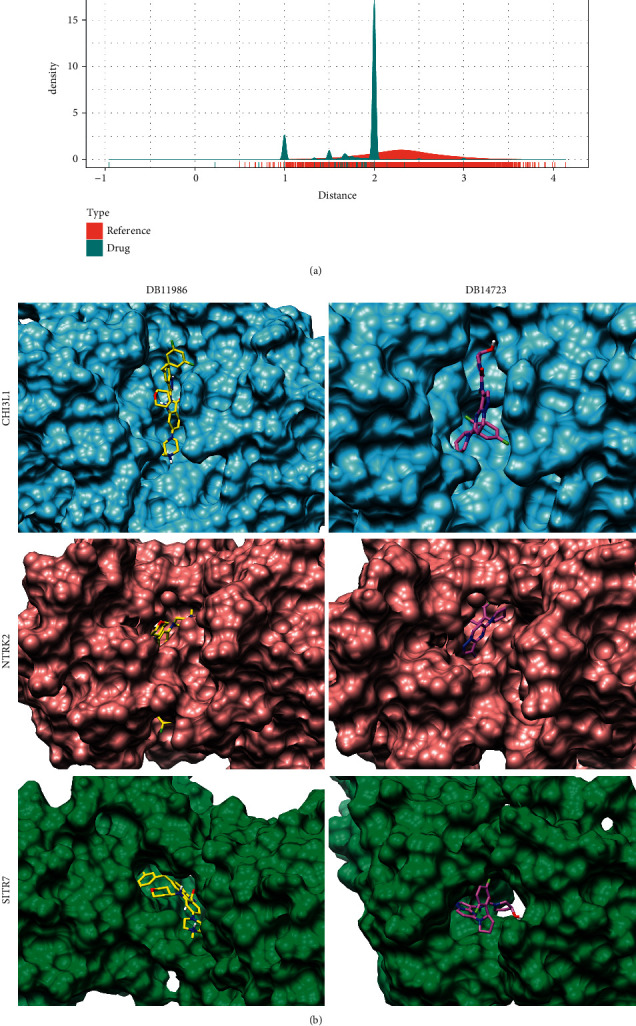
Potential drugs and drug-target analysis of key IDD genes. (a) The distribution of network-regulated distance between drug and disease key genes. (b) Interaction results of drugs DB11986 and DB14723 with CHI3L1, NTRK2, and SIRT7 proteins. Among them, CHI3L1 protein was added sky blue surface, NTRK2 protein was added rosy brown surface, and SIRT7 protein was added sea green surface. The drug DB11986 was displayed as yellow, and DB14723 was displayed as orchid.

To further determine the potential drug targets of key IDD genes, following Wang et al. [18], we determined the network distance between these 3 key genes and 5490 drugs on DrugBank (Figure 6(a)), and found that the distance between the three key genes and the drug was shorter than that of the random background. A total of two drugs were determined according to a global FDR < 0.05 (Table 2). Subsequently, the relationship between these two drugs and the three key IDD genes (SIRT7, NTRK2, CHI3L1) was further analyzed by molecular docking methods (Figure 6(b)). When drugs DB11986 and DB14723 were combined with CHI3L1 protein, both drugs could well bind to the active site of the protein and carried −9.7 kcal/mol and 10.0 kcal/mol, respectively. Such a high docking score indicated that these two molecules may have potential biological activity against CHI3L1 protein. When the two drugs bound to NTRK2 protein, the docking score was significantly reduced to −8.6 kcal/mol and −7.9 kcal/mol, respectively, though both of them bound to the active site. The drug DB11986 could be extended from the other side of the active site due to the molecular structure of the additive farm, but DB14723 was all embedded into NTRK2 protein for its relatively small molecular structure. Interestingly, when drugs DB11986 and DB14723 interacted with SIRT7 protein, the docking scores of the two drugs were significantly different. Among them, the docking score of DB11986 for SIRT7 was −9.5 kcal/mol, whereas that of DB14723 for SIRT7 was −7.9 kcal/mol. Such a significant difference also indicated that there may also be potential differences in the activity of these two drugs against SIRT7 protein. These results suggest that the different binding affinities of the two drugs to the three proteins could indicate the potential differences in interaction and biological activity.

### 2.6. Identification and Validation of IDD Biomarker

**Figure 7:**
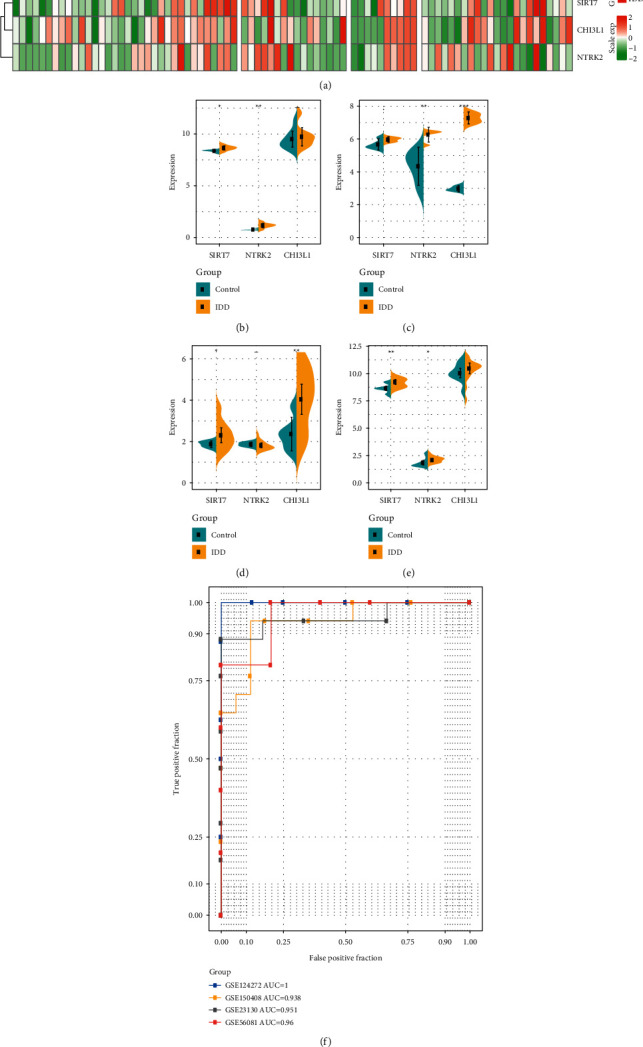
Identification and validation of IDD biomarker. (a) Heatmaps of expression profiles of SIRT7, NTRK2, CHI3L1 genes in the training set, test set, GSE150408 Dataset, and validation set. (b) Differential distribution of SIRT7, NTRK2, CHI3L1 genes in the GSE124272 dataset. (c) Differential distribution of SIRT7, NTRK2, CHI3L1 genes in the GSE56081 dataset. (d) Differential distribution of SIRT7, NTRK2, CHI3L1 genes in the GSE23130 dataset. (e) Differential distribution of SIRT7, NTRK2, CHI3L1 genes in the GSE150408 dataset. (f) Classification of ROC curve of the lncRNA diagnostic model in four datasets.

Markers related to IDD were further determined based on three disease-specific genes, we used GSE124272 as the training set, GSE56081 as the test set, and GSE23130 and GSE150408 as the external validation set. SIRT7, NTRK2, and CHI3L1 served as features in the training dataset to obtain their corresponding expression profiles. The heat map of expression profiles in each dataset demonstrated that SIRT7, NTRK2, and CHI3L1 were all highly expressed in the IDD group in different datasets (Figure 7(a)). After analyzing the expressions of the three genes in different datasets, we found that SIRT7 and NTRK2 genes were significantly overexpressed in GSE124272 (Figure 7(b)), that NTRK2 and CHI3L1 were significantly highly expressed in GSE56081 dataset (Figure 7(c)), that SIRT7 and CHI3L1 were significantly highly expressed in GSE23130 dataset (Figure 7(d)), and that SIRT7 and NTRK2 were significantly highly expressed in GSE150408 dataset (Figure 7(e)). Also, we added experimental validation, specifically, we collected tissues from five early IDD patients (III) and five advanced IDD patients (V) from The Third People's Hospital of Nanning and evaluated the expression differences of SIRT7, NTRK2, and CHI3L1 using RT-PCR, and as expected, they had a trend of higher expression in advanced IDD patients, with CHI3L1 and NTRK2 having a significant expression difference (Supplementary Figure S1). These findings suggested that the expression of a single gene in different datasets was easily disturbed by other factors. Therefore, we used the three genes as a panel to construct a SVM classification model. Tenfold cross-validation was used to test the model, and the classification accuracy was 100%, as all the 16 samples were correctly classified in the training dataset. The sensitivity of the model to IDD was 100%, the specificity was 100%, and the area under ROC curve (AUC) was 1.0. When using the GSE56081 dataset for verification, 9 out of 10 samples were correctly classified, with a classification accuracy of 90%, a model sensitivity to IDD of 80%, a specificity of 100%, and an area under ROC curve of 0.96. The GSE23130 dataset was further used for verification and accurately classified 19 samples out of 23, with a classification accuracy of 83.6%, a sensitivity of the model to IDD of 50%, a specificity of 94%, and area under ROC curve of 0.95. The GSE150408 dataset was further used for verification and accurately classified 27 samples out of 34, with a classification accuracy of 88.2%, a sensitivity of the model to IDD of 70.6%, a specificity of 79.4%, and an area under ROC curve of 0.94 (Figure 7(f)). These results indicated that the diagnostic prediction model based on SIRT7, NTRK2, and CHI3L1 could effectively distinguish IDD patients from control population; therefore, these genes could serve as reliable biomarkers for specific diagnosis of IDD.

## 3. Discussion

Low back pain (LBP) caused by intervertebral disc degeneration (IDD) is the most common musculoskeletal system disease [19]. IDD is the result of the interaction of many factors, including abnormal pressure load, inflammatory factors, cell aging, and related signal pathways, but the final result is the imbalance of extracellular matrix synthesis and catabolism [20]. In this study, the gene expression patterns between IDD and healthy samples were systematically analyzed, and two disease-related gene modules were identified by the weighted co-expression method. These genes were mainly enriched in neutrophil activation and regulation of cytokine-mediated signaling pathways, and extracellular matrix-related multiple biological pathways, suggesting that these modular genes have a strong biological correlation with IDD. Based on this, we constructed a protein interaction network and observed high-degree nodes with known IDD, and found that a higher correlation of related genes. Finally, IDD-related genes were introduced to establish a disease-specific network. Through the analysis of network topology, SIRT7, NTRK2, and CHI3L1 were finally identified as new IDD-specific genes, and these genes were significantly highly expressed in IDD samples.

Sirtuin 7 (SIRT7), which is a nicotinamide adenine dinucleotide (NAD+)-dependent histone deacetylase, is mainly located in the nucleus. SIRT7 is involved in a variety of cellular processes, including aging, DNA repair, tumorigenesis, and metabolism [21, 22]. SIRT7 is proven to be an important regulator of cartilage homeostasis and is involved in the development of OA [23]. SIRT7 expression is significantly downregulated in OA articular cartilage, which is consistent with autophagy gene expression; moreover, loss of SIRT7 accelerates type II collagen catabolism [24]. Neurotrophic receptor tyrosine kinase 2 (NTRK2) is a member of the neurotrophic receptor kinase (NTRK) family and a membrane-bound receptor. When neurotrophic proteins bind, members of the NTRK family and MAPK pathways are phosphorylated and give out signal through NTRK2, leading to cell differentiation. Jinhuai Hu et al. reported that NTRK2 is an oncogene, and its overexpression partially reverses the inhibitory effect of miR-22 on tumor proliferation and invasion [25]. Inflammation-related autocrine factor CHI3L1, which is tissue-specific and significantly upregulated during denaturation, protects IDD by promoting the Akt3 signaling pathway [8]. CHI3L1 can be expressed by a variety of cells, including chondrocytes, smooth muscle cells, and osteosarcoma cells, but its function is usually related to inflammation and tissue remodeling [26–28]. According to current studies, SIRT7 and NTRK2 have not been previously reported in IDD. The current study is the first to reveal the involvement of these two genes may be involved in the occurrence and development of IDD.

Entrectinib is an effective oral tyrosine kinase inhibitor of TrkA, TrkB, and TrkC (encoded by the genes neurotrophic tyrosine receptor kinase (NTRK) 1, 2, and 3, respectively). In a clinical study of 25 patients who had various malignancies containing NTRK, ROS1, or ALK gene fuses and received an effective dose of entrectinib, an overall response rate of 79% with significant tumor regression in all NTRK-altered tumors (including ETv6: NTRK3 translocation) [29] was observed. Larotrectinib is a selective inhibitor of neurotrophin receptor kinase (NTRK) and can be used to treat solid tumors carrying NTRK gene fusion [30, 31]. David S Hong et al. showed that among 159 patients with TRK fusion-positive cancer who received larotrectinib, 121 out of 153 evaluable patients showed an objective response (79%, 95% CI 72–85), and 24 (16%) showed a complete response (16%) [32]. As NTRK2 was confirmed as a prognostic gene for IDD in this study, we speculated that entrectinib and larotrectinib may relieve IDD through NTRK2.

Although we analyzed and verified the abnormal expression and functional role of genes in IDD from multiple data coalitions using bioinformatics techniques, some limitations of this study should be noted. Firstly, the sample lacked some clinical follow-up information; thus, we failed to consider factors such as the presence of other patient health conditions. Secondly, the results obtained only by bioinformatics analysis were insufficient, which required further experimental validation. Therefore, further genetic and experimental studies with larger sample sizes and experimental validation are needed.

## 4. Conclusion

In conclusion, in this study, we systematically analyzed the gene expression patterns in IDD and conducted a large-scale genome-wide study on the RNA expression profile to identify two gene modules closely related to IDD. Three new IDD-specific genes have been found for IDD through disease-association network mining, and the three genes were involved in a variety of important biological pathways. At the same time, we also discovered that entrectinib and larotrectinib may be effective in the treatment of IDD, which provides a target and reference for clinicians and biological experimentalists.

## 5. Materials and Methods

### 5.1. RNA Expression Profile

All gene expression profiles of human intervertebral disc degeneration were retrieved from the Gene Expression Omnibus (GEO) database (https://www.ncbi.nlm.nih.gov/geo/), and 4 datasets with a sample size of no less than 10, namely, GSE56081 [33], GSE124272 [34], GSE23130 [7], and GSE150408, were selected. Among them, there were 10 samples in GSE56081, including 5 samples from patients with IDD and 5 samples from the nucleus pulposus of normal control. The platform was Arraystar Human lncRNA microarray V2.0 (Agilent_033010 Probe Name version). The GSE124272 dataset contained of 8 IDD samples and 8 control samples on the Agilent-072363 SurePrint G3 Human GEV3 8 × 60 K Microarray 039494. The GSE23130 dataset contained a total of 23 samples on Affymetrix Human X3P Array.

The GSE56081 dataset is a lncRNA chip platform. The probe sequence of the GSE56081 dataset was aligned to the genome (GRCh38.p13, https://ftp.ebi.ac.uk/pub/databases/gencode/Gencode_human/release_39/gencode.v39.primary_assembly.annotation.gff3.gz) through the method of chip re-annotation to determine the transcript ID mapped by the probe. Each transcript cluster was assigned to Ensembl gene ID to obtain the matching relationship between probe and gene to acquire gene expression profile.

The specific process is as follows:

1. The matrix files expressing the sequence tags were downloaded to obtain the nucleic acid sequences of these probes.
2. The nucleic acid sequences of these probes were matched to the human genome library (ENCODE database, version 38, https://www.gencodegenes.org/human/) using SeqMap software [35]. The library requires sequence matches and no mismatches, and the corresponding chromosomal positions of the probes were obtained.
3. A total of 19873 re-annotated mRNA probes were obtained by simultaneously removing the presence of multiple matching probes.

Finally, for all the expression profiles, the probe was mapped to the gene, and when multiple probes were mapped to the same gene, the median value was taken as the expression value of the gene. To enlarge the sample size of the dataset, we combined the GSE56081 and GSE124272 datasets, combat function of R software package SVA [36] was used to remove the batch effect to obtain a new expression profile, and the GSE23130 dataset served as an external independent verification queue.

### 5.2. Weighted Co-Expression Network Analysis

After merging the datasets of GSE56081 and GSE124272 and removing the batch effect, the weighted co-expression module was constructed using the gene expression profile. Specifically, the RNA expression data profile of the genes was used to examine whether the samples and genes were qualified. Then, we used the WGCNA [37] package in R to construct a scale-free co-expression network for the genes. The Pearson's correlation matrices and average linkage method were performed for pair-wise. Then, a weighted adjacency matrix was constructed using a power function *A*_*mn*_=|*C*_*mn*_|^*β*^ (*C*_*mn*_ = Pearson's correlation between gene *m* and gene *n*; *A*_*mn*_ = adjacency between gene *m* and gene *n*). *β*, which is a soft-thresholding parameter, emphasizes strong correlations between gene and indicates weak correlations. After determining the power of *β*, the adjacency was transformed into a topological overlap matrix (TOM) for measuring the network connectivity of a gene, which was defined as the ratio of sum of its adjacency to all other genes, and then, the corresponding dissimilarity (1-TOM) was calculated. To classify genes with similar expression profiles into gene modules, average linkage hierarchical clustering was performed according to the TOM-based dissimilarity measured with a minimum size (gene group) of 30 for the gene dendrogram. To further analyze the module, we calculated the dissimilarity of module eigen gene, determined a cut line for module dendrogram, and merged some modules.

### 5.3. Identification of Co-Expression Modules Associated with IDD

We defined the module related to the occurrence of IDD as the IDD Module. Specifically, the correlation between ME and IDD features was calculated to identify the relevant modules with significance *P* < 0.05. Further analysis on the distribution differences of each module's feature vectors in IDD and control group was performed to select the modules with significant FDR < 0.05. Also, we obtained known IDD-related gene (IDDRG) sets from the DisGeNET [14] database, analyzed the intersection of genes and IDDRGs in each Module, evaluated the enrichment significance of IDDRG by hypergeometric test, and selected the modules with significantly rich IDDRGs as the final IDD Module.

### 5.4. Functional Enrichment Analyses

Gene Ontology (GO) and Kyoto Encyclopedia of Genes and Genomes (KEGG) pathway enrichment analysis was performed using the R package clusterProfiler [38] for screening genes associated with the IDD Module, so as to identify over-represented GO terms in three categories (biological processes, molecular function, and cellular component) and KEGG pathway. For both analyses, a FDR of <0.05 was considered to denote statistical significance.

### 5.5. Construction of IDD-Related Protein Interaction Network

In the IDD Module, the correlation between the genes in the Module and the feature vectors of the Module was calculated to select gene set with the correlation coefficient greater than 0.7. The classification performance of each gene expression in IDD and control group was further analyzed, and the gene set with AUC greater than 0.8 was determined as the final core gene set of IDD Module. These gene sets were mapped to the STRING v11.0 [16] database to obtain the interaction data among these genes, and an IDD-related protein interaction network (IDDPPI) was established. Visual analysis was performed using cytoscope [39], and the degree of nodes in the protein interaction network was used as the rank. GSEA [40] enrichment analysis was employed to obtain significantly enriched KEGG pathways to evaluate network function.

### 5.6. Construction of IDDRG-IDDPPI-Related Network

The genes in IDDRG and IDDPPI (IDDPPIG) were mapped to the STING V11.0 [16] database to construct a protein interaction network. The degree distribution of each IDDPPIG and IDDRG in the network was further analyzed. The significance of each IDDPPIG enriched by IDDRG and the proportion of IDDPPIG gene interaction were calculated using a hypergeometric test to analyze the network characteristics of IDDPPIG and IDDRG, and the average shortest path between two IDDPPIG or between IDDPPIG and IDDRG was compared. The multiple relationship distribution of the average shortest path between two IDDPPIG genes and between an IDDRG and an IDDPPIG gene was calculated. Based on the above characteristics, the random perturbation method was used to establish a random network as the background, and the significant genes were selected as the new key genes of IDD (IDDG).

### 5.7. IDDG and Drug-Target Network Construction

To examine the potential drug effects of IDDG, the relationship between drugs and drug-target genes was obtained from DrugBank v5.1.7 database [41], and a total of 16196 drug-gene interaction data were identified. These drug-target genes and IDDG genes were mapped to the STRING V11.0 [16] database to obtain gene interaction information, and a drug-gene-IDDG network was constructed. As previously described by Wang et al. [18], the shortest path of drugs to IDDG was calculated for identifying potentially related drugs to IDDG.

Specifically, we calculated the proximity of the drug to IDDG. In this case, we can give S, the IDD-related gene set IDDG; *D*, the degree of IDD-related gene set nodes in PPI; and *T*, drug-target gene collection. Distance *D* (s, t) is the shortest path between *s* node and *T* node (where *S* ∈ *S* is IDD-related gene; *T* ∈ *T* is drug-target gene), and the calculation method is as follows:

(1) $$\begin{array}{c} d\left(S,T\right)=\frac{1}{\left|T\right|}\sum_{t\in T}\underset{s\in S}{\min}\left(d\left(s,t\right)+\omega \right), \end{array}$$

where *ω* is the weight of the target gene. If the target gene is a gene in the IDD-related gene set, the calculation method is *ω* = −ln (*D *+* *1); otherwise, *ω* = 0.

We generated the simulated reference distance distribution corresponding to the drug. To put it simply, a group of protein nodes were randomly selected in the network as the simulated drug target, and the number of nodes was the same as the target size (denoted as R). Next, the distances *d* (*S*, *R*) between these simulated drug targets (representing the simulated drug) and DMEGs were calculated. After 1000 random repeats, the simulated reference distributions were generated. At the same time, the mean and standard deviation of the *μ*d (*S*, *R*) and *σd* (*S*, *R*) reference distributions and the corresponding actual observed distances were converted into standardized scores, that is, proximity *Z*:

(2) $$\begin{array}{c} z\left(S,T\right)=\frac{d\left(S,T\right)-\mu_{d\left(S,R\right)}}{\sigma_{d\left(S,R\right)}}. \end{array}$$

Finally, the shortest path to IDDG was significantly higher for the drug than for the background drug according to the simulated reference distance distribution. The degree of binding between IDDG and drugs was evaluated by molecular docking.

### 5.8. Establishment of IDD Diagnostic Prediction Model and Evaluation of Model Prediction Performance

IDDG was used to construct a diagnostic prediction model based on support vector machine (SVM) classification [42] to predict the IDD and control samples. SVM, which is a supervised machine learning algorithm model, analyzes data, and identifies patterns. A SVM creates a hyperplane, in high or infinite dimensions, and can be used for classification and regression. Given a set of training samples in which each marker belongs to two classes, an SVM training algorithm builds a model that assigns new instances to one class or another, making it an improbabilistic binary linear classification.

In this study, GSE124272 was the training set, GSE56081 was the test set, and GSE23130 was the external verification set. The model was constructed in the training dataset, and the classification ability of the model was verified by the tenfold cross-validation method. The established model was then used to predict the samples in the test set and validation dataset. The predictive ability of the model was evaluated using area under the ROC curve (AUC), and the sensitivity and specificity of the model for IDD prediction were analyzed.

### 5.9. Statistical Analysis

Ggplot2 of R software was used for visualization, and heatmap was used to draw heat maps. Fisher's exact test was used for multigroup comparison, and significance was defined as *P* < 0.05. The Benjamini method was used for multiple test correction to obtain FDR. All of these analyses are performed in R 3.4.3.

## Figures and Tables

**Table 1:** Network characteristics of 3 key genes.

Symbol	IDDRG Count	IDDRG Count p value	IDDRG ratio	IDDRG ratio p value	Shortest ratio	Shortest Ratio_p value	IDDRG enrichment p value
SIRT7	16	0.000609	0.94	0.024	0.76	0.01	4.93E-28
NTRK2	21	3.04E-06	0.87	0.041	0.77	0.015	5.33E-35
CHI3L1	18	8.84E-05	0.86	0.048	0.76	0.01	9.30E-30

**Table 2:** Potential drugs for key genes.

Drug_id	Drug name	Distances	P value	FDR
DB11986	Entrectinib	0.2238934	5.219693e-06	2.865090e-02
DB14723	Larotrectinib	-0.9601777	2.416713e-12	1.326775e-08

## Data Availability

The data that support the findings of this study are available from the corresponding author upon reasonable request.

## Supplementary Materials

Supplementary Materials
*Figure S1.* The mRNA expression differences of SIRT7, NTRK2, and CHI3L1 early IDD patients (III) and advanced IDD patients (V).
