# Supplementary figures and images for: Comprehensive Network Analysis Identified SIRT7, NTRK2, and CHI3L1 as New Potential Markers for Intervertebral Disc Degeneration
Source: J Oncol. 2022 Feb 12;2022:4407541. doi: 10.1155/2022/4407541 (PMC8858045; doi:10.1155/2022/4407541)

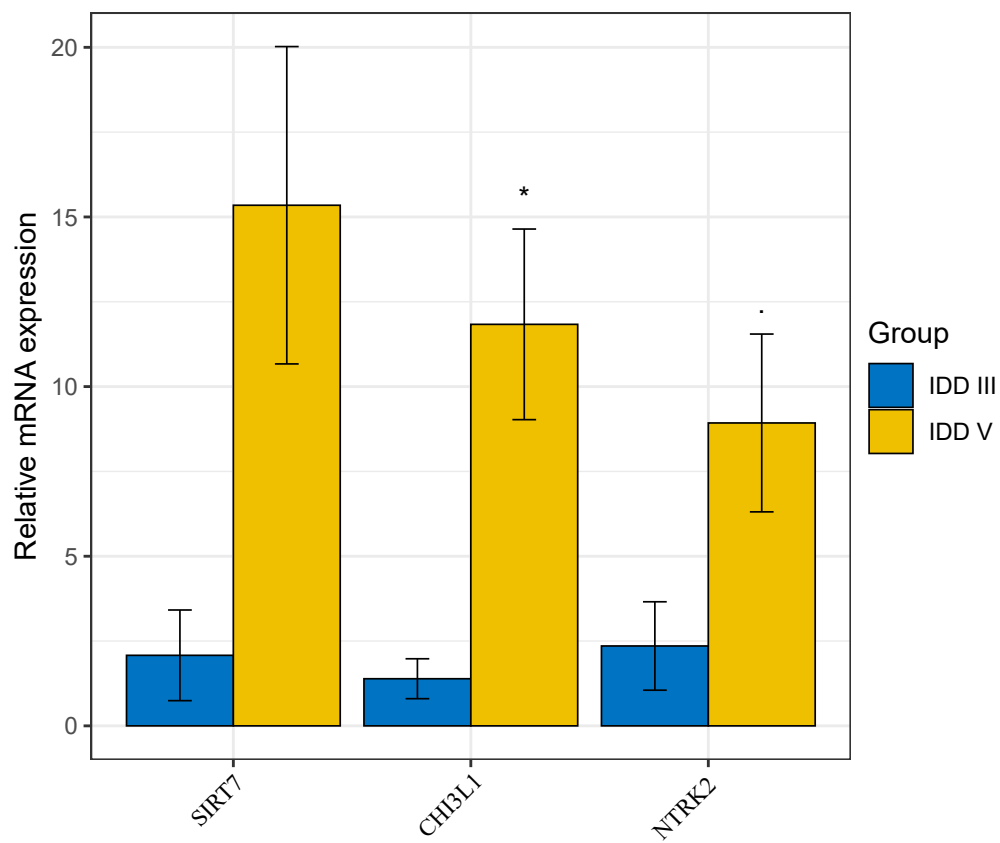

Supplement: Supplementary Materials — Figure S1. The mRNA expression differences of SIRT7, NTRK2, and CHI3L1 early IDD patients (III) and advanced IDD patients (V). [file 4407541.f1.pdf]
